# Porcine reproductive and respiratory syndrome virus 2 hijacks CMA-mediated lipolysis through upregulation of small GTPase RAB18

**DOI:** 10.1371/journal.ppat.1012123

**Published:** 2024-04-12

**Authors:** Guo-Li Li, Ying-Qian Han, Bing-Qian Su, Hai-Shen Yu, Shuang Zhang, Guo-Yu Yang, Jiang Wang, Fang Liu, Sheng-Li Ming, Bei-Bei Chu

**Affiliations:** 1 College of Veterinary Medicine, Henan Agricultural University, Zhengzhou, Henan Province, China; 2 Key Laboratory of Animal Biochemistry and Nutrition, Zhengzhou, Henan Province, Ministry of Agriculture and Rural Affairs of the People’s Republic of China; 3 Key Laboratory of Veterinary Biotechnology of Henan Province, Zhengzhou, Henan Province, China; 4 Ministry of Education Key Laboratory for Animal Pathogens and Biosafety, Zhengzhou, Henan Province, China; 5 International Joint Research Center of National Animal Immunology, Henan Agricultural University, Zhengzhou, Henan Province, China; 6 Longhu Advanced Immunization Laboratory, Zhengzhou, Henan Province, China; Georgia State University, UNITED STATES

## Abstract

RAB GTPases (RABs) control intracellular membrane trafficking with high precision. In the present study, we carried out a short hairpin RNA (shRNA) screen focused on a library of 62 RABs during infection with porcine reproductive and respiratory syndrome virus 2 (PRRSV-2), a member of the family *Arteriviridae*. We found that 13 RABs negatively affect the yield of PRRSV-2 progeny virus, whereas 29 RABs have a positive impact on the yield of PRRSV-2 progeny virus. Further analysis revealed that PRRSV-2 infection transcriptionally regulated RAB18 through RIG-I/MAVS-mediated canonical NF-κB activation. Disrupting RAB18 expression led to the accumulation of lipid droplets (LDs), impaired LDs catabolism, and flawed viral replication and assembly. We also discovered that PRRSV-2 co-opts chaperone-mediated autophagy (CMA) for lipolysis via RAB18, as indicated by the enhanced associations between RAB18 and perlipin 2 (PLIN2), CMA-specific lysosomal associated membrane protein 2A (LAMP2A), and heat shock protein family A (Hsp70) member 8 (HSPA8/HSC70) during PRRSV-2 infection. Knockdown of HSPA8 and LAMP2A impacted on the yield of PRRSV-2 progeny virus, implying that the virus utilizes RAB18 to promote CMA-mediated lipolysis. Importantly, we determined that the C-terminal domain (CTD) of HSPA8 could bind to the switch II domain of RAB18, and the CTD of PLIN2 was capable of associating with HSPA8, suggesting that HSPA8 facilitates the interaction between RAB18 and PLIN2 in the CMA process. In summary, our findings elucidate how PRRSV-2 hijacks CMA-mediated lipid metabolism through innate immune activation to enhance the yield of progeny virus, offering novel insights for the development of anti-PRRSV-2 treatments.

## Author summary

Two species of arteriviruses, a family of RNA viruses, can cause severe reproductive disorders and respiratory diseases in infected piglets that result in significant economic losses in the swine industry. The respective viruses conventionally known as PRRSV-1 and PRRSV-2 exhibit high genetic variability and immune evasion capabilities, which complicate the development of effective vaccines and control measures. In this study, we have described a unique mechanism by which PRRSV-2 infection stimulates RAB18 expression through RIG-I/MAVS-mediated canonical NF-κB activation. RAB18 then recruits HSPA8 and PLIN2 for CMA-mediated degradation and subsequent lipolysis, facilitating viral replication and assembly. Our study provides new insights into the interplay between innate immunity, RAB18, and the CMA-mediated lipophagy pathway during RNA virus infection.

## Introduction

The family *Arteriviridae* are positive-stranded RNA viruses including more than 20 species whose infection in natural hosts may range from asymptomatic to persistent and be associated with acute disease including respiratory syndrome, abortion, or lethal hemorrhagic fever [1]. Three species *Betaarterivirus suid 1*, *Betaarterivirus suid 2* and *Alphaarterivirus equid* infect domestic animals, and they include two porcine reproductive and respiratory syndrome viruses (PRRSV-1 and PRRSV-2) and equine arteritis virus, respectively [2, 3]. Infections with PRRSV-1 and PRRSV-2 are of particular concern for the swine industry due to the economic impact of its complications resulting in severe respiratory disease in pigs [4, 5]. Infection with both porcine arteriviruses primarily targets the fully differentiated porcine alveolar macrophage (PAM) [6]. Among the numerous cell lines tested, only the African green monkey kidney cell line MA-104 and its derivatives, such as MARC-145, demonstrate complete permissiveness to the yield of PRRSV-1 and PRRSV-2 progeny virus *in vitro* [7]. Two PRRSV species have evolved several strategies to evade the immune response, which include inhibiting cytokine production and modulating the expression of major histocompatibility complex class I molecules and costimulatory molecules [8, 9]. Understanding the interactions between these porcine arteriviruses and their host is crucial for the development of effective vaccines and therapies.

RAB guanosine triphosphatases (GTPases) are a family of small GTPases that regulate intracellular membrane trafficking in eukaryotic cells [10]. They are involved in vesicle formation, transport, and fusion with target membranes [11]. There are more than 60 members of the RAB family in humans, each with its own unique localization and function [12]. Many viruses have evolved mechanisms to interact with RABs to manipulate the host’s intracellular trafficking machinery for their own replication and propagation [13]. For example, the rabies virus hijacks the host cell’s endocytic pathway through interaction with RAB5, enabling entry and infection of cells [14]. The hepatitis C virus has been shown to utilize several RABs, including RAB5, RAB7, and RAB11, to facilitate its replication and assembly [15]. Herpes simplex virus type 1 infection leads to the formation of RAB27a-positive vesicles that transport viral particles to the cell periphery for efficient viral spread [16]. Rab18 promotes dengue virus infection by directing fatty acid synthase toward the locations of viral replication [17]. Understanding the interactions between PRRSV-1 and PRRSV-2, and RABs could help in the development of strategies to control these arteriviruses.

Autophagy is a cellular process that involves the degradation and recycling of damaged or unnecessary cellular components [18]. The process of CMA involves the recognition of a specific targeting motif on target proteins by the cytosolic chaperone protein, heat shock cognate 71 kDa protein (often referred to as heat shock protein family A (Hsp70) member 8, or HSPA8). Once recognized, the target protein is unfolded and translocated across the lysosomal membrane facilitated by a complex of lysosome-associated membrane protein 2A (LAMP2A). Upon entering the lysosomal lumen, the target protein is degraded by lysosomal proteases. Recent studies have shown that CMA plays a role in the regulation of lipid droplets (LDs) [19], which are intracellular organelles that store triglycerides and cholesterol esters [20]. In CMA, LDs-associated proteins are recognized by HSPA8 and delivered to lysosome for degradation [21]. This process can regulate lipid storage and mobilization, as well as lipolysis and lipogenesis [21]. However, the role of CMA-mediated lipolysis in the yield of PRRSV-2 progeny virus has not been documented.

In this study, we performed short hairpin RNA (shRNA) screening using a targeted library of RAB genes during PRRSV-2 infection and found that most RAB genes were involved in the yield of PRRSV-2 progeny virus. We further demonstrated that PRRSV-2 hijacked CMA-mediated lipolysis through the upregulation of RAB18 via the canonical nuclear factor-kappa B (NF-κB) pathway. These findings provide insights into the mechanism by which PRRSV-2 utilizes lipolysis upon the activation of innate immunity.

## Results

### Screening for RAB family proteins that regulate the yield of PRRSV-2 progeny virus

**Fig 1:**
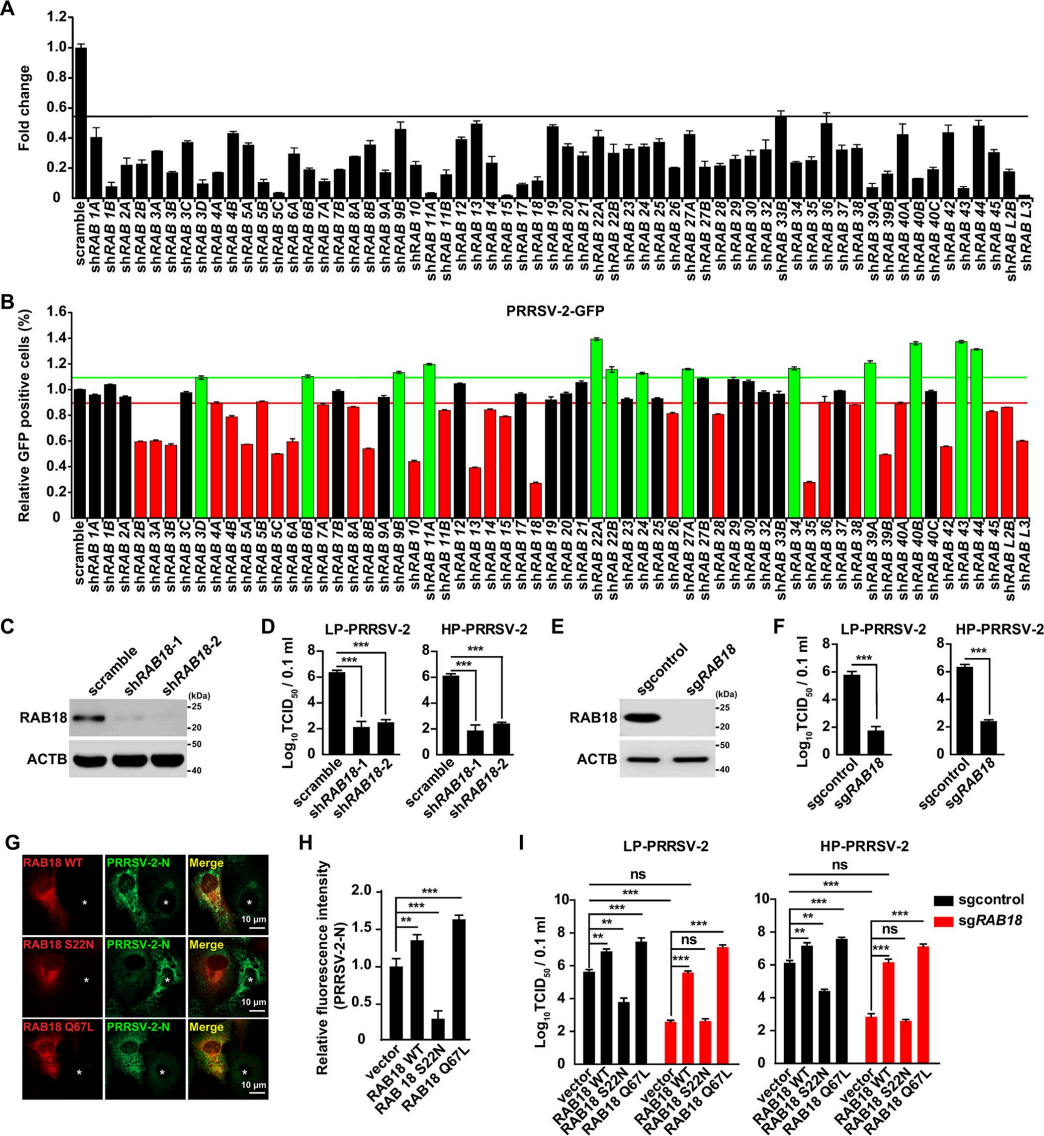
Identifying regulatory RAB proteins in the yield of PRRSV-2 progeny virus. (A) The mRNA levels of the indicated RABs in sh*RABs* MARC-145 cells were analyzed by qRT-PCR analysis. Values below the black line are statistically significance. (B) sh*RABs* MARC-145 cells were infected with PRRSV-2-GFP (MOI = 10) for 36 h. GFP-positive cells were analyzed by flow cytometry. Values above the green line (green bar) and below the red line (red bar) are statistically significance. (C) Immunoblot analysis of RAB18 and ACTB in scramble, sh*RAB18*-1, and sh*RAB18*-2 MARC-145 cells. (D) Scrambled, sh*RAB18*-1, and sh*RAB18*-2 MARC-145 cells were infected with LP-PRRSV-2 (MOI = 10) and HP-PRRSV-2 (MOI = 10) for 36 h. Viral titers were assessed by the TCID_50_ assay. ***P < 0.001. (E) Immunoblot analysis of RAB18 and ACTB in sgcontrol and sg*RAB18* MARC-145 cells. (F) Sgcontrol and sg*RAB18* MARC-145 cells were infected with LP-PRRSV-2 (MOI = 10) and HP-PRRSV-2 (MOI = 10) for 36 h. Viral titers were assessed by the TCID_50_ assay. ***P < 0.001. (G) MARC-145 cells were transfected with plasmids encoding RAB18-mCherry WT, RAB18-mCherry S22N, and RAB18-mCherry Q67L for 12 h and then infected with LP-PRRSV-2 (MOI = 10) for 36 h. PRRSV-2-N was detected by immunofluorescence analysis. Asterisks indicate non-transfected cells. Scale bar: 10 μm. (H) Quantification of the relative fluorescence intensity of PRRSV-2-N from (G) (n = 20). **P < 0.01, ***P < 0.001. (I) Sgcontrol and sg*RAB18* MARC-145 cells were transfected with plasmids encoding RAB18-FLAG WT, RAB18-FLAG S22N, and RAB18-FLAG Q67L for 12 h and then infected with LP-PRRSV-2 (MOI = 10) and HP-PRRSV-2 (MOI = 10) for 36 h. Viral titers were assessed by the TCID_50_ assay. **P < 0.01, ***P < 0.001. ns, not significance.

To investigate the role of RABs in the yield of PRRSV-2 progeny virus, we used lentivirus-mediated RNA interference (RNAi) to knock down RAB genes by the infection of MARC-145 cells. Sixty-two RAB genes were efficiently knocked down, as indicated by quantitative real-time polymerase chain reaction (qRT-PCR) analysis (Fig 1A), and no obvious cytotoxicity was detected in these cells (S1A Fig). We then infected these cells with PRRSV-2-green fluorescent protein (GFP) and analyzed progeny virus production by flow cytometry. At 36 hours post-infection, we found that the yield of PRRSV-2-GFP progeny virus was negatively regulated by 13 RABs (RAB3D, RAB6B, RAB9B, RAB11A, RAB22A, RAB22B, RAB24, RAB27A, RAB34, RAB39A, RAB40B, RAB43, and RAB44) and positively regulated by 29 RABs (RAB2B, RAB3A, RAB3B, RAB4A, RAB4B, RAB5A, RAB5B, RAB5C, RAB6A, RAB7A, RAB8A, RAB8B, RAB10, RAB11B, RAB13, RAB14, RAB15, RAB18, RAB26, RAB28, RAB35, RAB36, RAB38, RAB39B, RAB40A, RAB42, RAB45, RABL2B, and RABL3) (Fig 1B). Notably, knockdown of RAB18 most significantly affected the yield of PRRSV-2-GFP progeny virus (Fig 1B). Several reports have indicated that RAB18 is involved in the replication of classical swine fever virus [22], dengue virus [17], and hepatitis C virus [23]. However, no report has yet suggested that RAB18 participates in PRRSV-2 progeny virus production. Therefore, we chose RAB18 for further investigation.

To confirm the role of RAB18 in the yield of PRRSV-2 progeny virus, we generated two types of MARC-145 cells (sh*RAB18*-1 and sh*RAB18*-2) that stably expressed low levels of RAB18 (Fig 1C). Although RAB18 knockdown did not affect cell growth (S1B Fig), it impaired the replication of both low- and high-pathogenic strains of PRRSV-2 (LP-PRRSV-2 and HP-PRRSV-2, respectively), as evidenced by tissue culture infective dose (TCID_50_) assay of viral titers (Fig 1D). We also confirmed that sg*RAB18* cells, generated by CRISPR/Cas9 technology (Fig 1E), exhibited no cytotoxicity but showed reduced the yield of both PRRSV-2 strains (Figs 1F and S1C), consistent with the phenotype of the sh*RAB18* lines. These results confirm that RAB18 plays a part in the yield of PRRSV-2 progeny virus.

We next examined whether the GTPase activity of RAB18 was required for the yield of PRRSV-2 progeny virus. We constructed plasmids expressing wild-type (WT) RAB18, an activated mutant of RAB18 (Q67L) [24], and a GDP-locked mutant of RAB18 (S22N) [24]. Cell viability was not affected by the overexpression of these three RAB18 variants (S1D Fig). Immunofluorescence analysis revealed that cells expressing RAB18 WT or RAB18 Q67L exhibited greater intensity of the PRRSV-2 nucleocapsid (N) protein compared to control cells (Fig 1G and 1H). However, RAB18 S22N inhibited the expression of the N protein (Fig 1G and 1H), suggesting that GTPase activity of RAB18 is required for the yield of PRRSV-2 progeny virus. To investigate the impact of RAB18 WT, S22N, and Q67L on the yield of PRRSV-2 progeny virus, we introduced them into both control and RAB18 knockout cells and measured the viral titer using a TCID_50_ assay. Both PRRSV-2 strains’ infectivity was enhanced by the overexpression of RAB18 WT and Q67L in control cells, whereas overexpression of RAB18 S22N impeded the yield of PRRSV-2 progeny virus (Fig 1I). Introduction of RAB18 WT and Q67L into sg*RAB18* cells effectively restored the yield of PRRSV-2 progeny virus; however, the RAB18 S22N variant did not have the same effect (Fig 1I). These data indicate that RAB18 is critical for PRRSV-2 replication.

### The RIG-I/MAVS/NF-κB signaling pathway mediates RAB18 upregulation during PRRSV-2 infection

**Fig 2:**
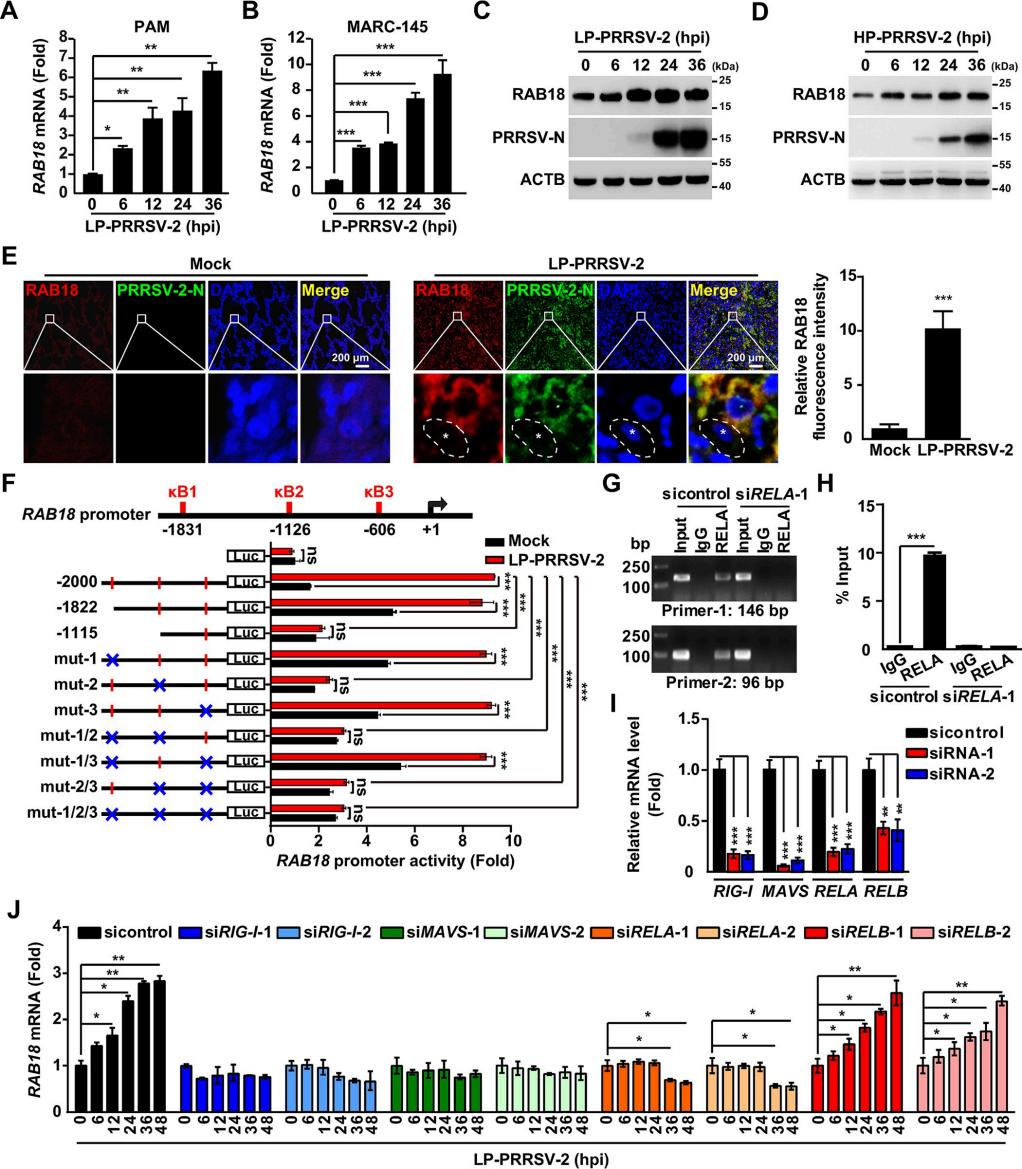
The RIG-I/MAVS/RELA cascade mediates RAB18 upregulation during PRRSV-2 infection. (A and B) PAM (A) and MARC-145 (B) cells were infected with LP-PRRSV-2 (MOI = 10) for 0–36 h. The mRNA levels of RAB18 were analyzed by qRT-PCR. *P < 0.05, **P < 0.01, ***P < 0.001. (C and D) MARC-145 cells were infected with LP-PRRSV-2 (MOI = 10, C) and HP-PRRSV-2 (MOI = 10, D) for 0–36 h. RAB18 and ACTB were analyzed by immunoblot analysis. (E) Immunohistochemistry for RAB18 and PRRSV-2-N in mock-infected or LP-PRRSV-2-infected porcine lungs (left). Asterisks indicate non-infected cells. Scale bar: 200 μm. The quantification of relative fluorescence intensity of PRRSV-2-N is shown on the right (n = 20). ***P < 0.001. (F) MARC-145 cells were transfected with the indicated RAB18-LUC plasmids for 12 h and then mock-infected or infected with LP-PRRSV-2 (MOI = 10) for 24 h. RAB18 promoter activities were assessed by dual-luciferase reporter assay. ***P < 0.001. ns, not significance. (G) MARC-145 cells were transfected with sicontrol or si*RELA*-1 for 24 h, followed by infection with LP-PRRSV-2 (MOI = 10) for 24 h. ChIP assays were performed with IgG or an antibody against RELA. The RAB18 promoter was amplified by primer-1 (146 bp) and primer-2 (96 bp). (H) The treatment of MARC-145 cells was as described in (G). ChIP assays were performed with IgG or an antibody against RELA. ***P < 0.001. (I) MARC-145 cells were transfected with sicontrol, siR*IG-I*-1, si*RIG-I*-2, si*MAVS*-1, si*MAVS*-2, si*RELA*-1, si*RELA*-2, si*RELB*-1, and si*RELB*-2 for 36 h. The mRNA levels of RIG-I, MAVS, RELA, and RELB were analyzed by qRT-PCR analysis. ***P < 0.001. (J) MARC-145 cells were transfected with sicontrol, siR*IG-I*-1, si*RIG-I*-2, si*MAVS*-1, si*MAVS*-2, si*RELA*-1, si*RELA*-2, si*RELB*-1, and si*RELB*-2 for 24 h, then infected with LP-PRRSV-2 (MOI = 10) for 0–48 h. The mRNA levels of RAB18 were analyzed by qRT-PCR analysis. *P < 0.05, **P < 0.01.

We then investigated if RAB18 expression was regulated by PRRSV-2 infection. Notably, PRRSV-2 infection resulted in increased levels of both RAB18 mRNA and protein in PAM and MARC-145 cells, as evidenced by qRT-PCR and immunoblot analysis (Fig 2A–2D). Furthermore, we observed RAB18 expression in mock-infected or PRRSV-2-infected porcine lungs using immunofluorescence analysis. Cells positive for PRRSV-2-N protein exhibited an elevated RAB18 signal compared to negative cells (Fig 2E). These findings indicate that RAB18 is upregulated during PRRSV-2 infection.

To investigate how PRRSV-2 infection transcriptionally enhanced *RAB18* expression, we analyzed the *RAB18* promoter and discovered three consensus binding sites for the transcription factor NF-κB (Fig 2F). Promoter mutation analysis using dual-luciferase reporter assays revealed that the second NF-κB binding site at position -1126 in the *RAB18* promoter was crucial for the PRRSV-2-induced upregulation of RAB18 (Fig 2F). To confirm the role of NF-κB in *RAB18* expression, we conducted chromatin immunoprecipitation (ChIP) assays with a RELA-specific antibody (the canonical subunit of NF-κB) to determine whether RELA bound to the RAB18 promoter. As shown in Fig 2G and 2H, RELA was recruited to the *RAB18* promoter. However, the knockdown of *RELA* hindered the association of RELA with the *RAB18* promoter (Fig 2G and 2H). These findings demonstrate that the canonical NF-κB pathway is responsible for the upregulation of RAB18 during PRRSV-2 infection.

The retinoic acid-inducible gene 1 (RIG-I) and mitochondrial antiviral signaling protein (MAVS) are crucial cytoplasmic pathogen recognition receptors that play a role in identifying RNA viruses and activating the NF-κB pathway [25]. Hence, we hypothesized that PRRSV-2 infection upregulates RAB18 expression through the RIG-I/MAVS/NF-κB pathway. To test this hypothesis, we utilized small interfering RNA (siRNA) to block the expression of *RIG-I*, *MAVS*, *RELA*, and *RELB* (the non-canonical subunit of NF-κB) (Fig 2I), with no evident cytotoxicity and enhanced viral replication observed in these cells (S1E and S1F Fig). qRT-PCR analysis revealed that knockdown of *RIG-1*, *MAVS*, and *RELA* prevented the upregulation of RAB18 during PRRSV-2 infection; however, interference with *RELB* did not produce the same effect (Fig 2J). Overall, our data indicate that RAB18 is upregulated by the RIG-I/MAVS-mediated canonical NF-κB pathway during PRRSV-2 infection.

### PRRSV-2 exploits RAB18 for lipolysis and efficient viral replication and assembly

**Fig 3:**
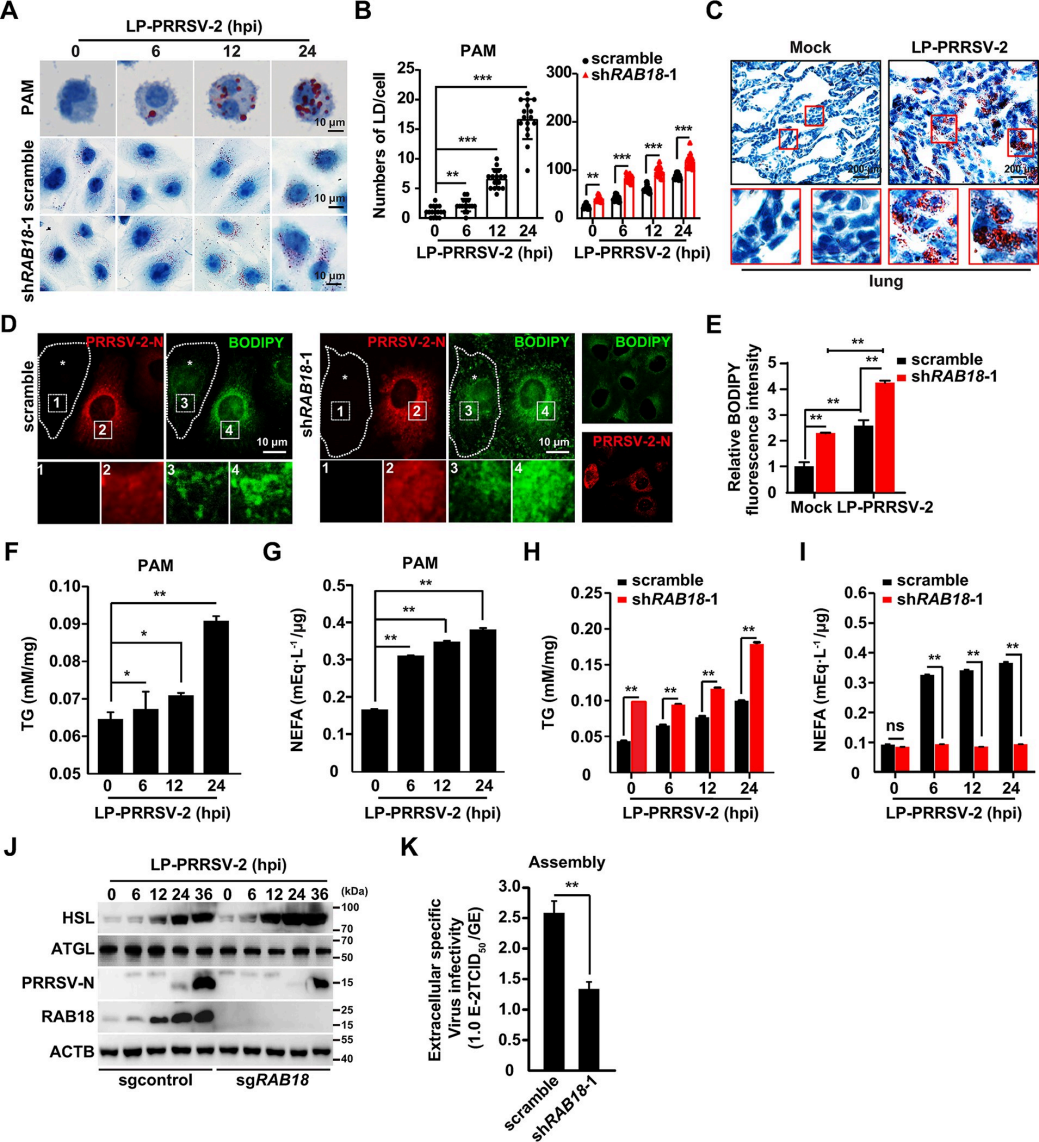
RAB18 silencing leads to increased LDs accumulation and disruption of viral replication and assembly. (A) PAM, scramble and sh*RAB18*-1 MARC-145 cells were infected with LP-PRRSV-2 (MOI = 10) for 0–24 h. LDs were analyzed by Oil Red O staining. Scale bar: 10 μm. (B) Quantification of LDs numbers per cell from (A) by ImageJ (n = 16). **P < 0.01, ***P < 0.001. (C) Oil Red O staining of mock-infected or LP-PRRSV-2-infected porcine lungs. Scale bar: 200 μm. (D) Scramble and sh*RAB18*-1 MARC-145 cells were infected with LP-PRRSV-2 (MOI = 10) for 24 h. PRRSV-2-N was detected by immunofluorescence analysis and LDs were examined by BODIPY staining. Uninfected cells, indicated by asterisks, were outlined with dashed lines. Scale bar: 10 μm. (E) Quantification of relative BODIPY fluorescence intensity in cells from (D) by ImageJ (n = 16). **P < 0.01. (F and G) PAM were infected with LP-PRRSV-2 (MOI = 10) for 0–24 h. TG (F) and NEFA (G) were quantified using biochemical kits. *P < 0.05, **P < 0.01. (H and I) Scramble and sh*RAB18*-1 MARC-145 cells were infected with LP-PRRSV-2 (MOI = 10) for 0–24 h. TG (H) and NEFA (I) were quantified using biochemical kits. **P < 0.01. ns, no significance. (J) Sgcontrol and sg*RAB18* MARC-145 cells were infected with LP-PRRSV-2 (MOI = 10) for 0–36 h. HSL, ATGL, PRRSV-2-N, RAB18, and ACTB were analyzed by immunoblot analysis. (K) Scramble and sh*RAB18*-1 MARC-145 cells were infected with LP-PRRSV-2 (MOI = 10) for 36 h. Viral assembly in the supernatants was determined by comparing the infectious titers (TCID_50_/mL) with the total PRRSV-2 genome equivalents (GE). **P < 0.01.

It has been observed that RAB18 is involved in LDs metabolism [26]. We hypothesized that PRRSV-2 upregulates RAB18 to utilize LDs for viral replication. Oil Red O staining in PAM and MARC-145 cells revealed that PRRSV-2 infection increased the number of LDs (Fig 3A and 3B). Conversely, knockdown of RAB18 inhibited LDs degradation, as evidenced by the greater number of LDs in sh*RAB18*-1 cells compared to control cells (Fig 3A and 3B). Increased LDs numbers were also noted in PRRSV-2-infected lung sections stained with Oil Red O compared to mock-infected sections (Fig 3C). BODIPY (Boron-Dipyrromethene) is a fluorescent dye known for its high affinity and fluorescence properties in neutral lipids and lipid droplets and is commonly used for staining these substances [27]. Furthermore, we assessed LDs in scramble and sh*RAB18*-1 MARC-145 cells by BODIPY staining and immunofluorescence of the PRRSV-2-N protein. As shown in Fig 3D and 3E, scramble cells exhibited weaker BODIPY fluorescence intensity than sh*RAB18*-1 cells in the absence of PRRSV-2-N expression. Scramble cells that were positive for PRRSV-2-N displayed a strong BODIPY signal, which was further enhanced by RAB18 knockdown (Fig 3D and 3E).

We then measured the lipid content in PAM and MARC-145 cells in response to PRRSV-2 infection. Significant increases in cellular triglyceride (TG) and non-esterified (free) fatty acids (NEFA) were observed in PRRSV-2-infected PAM and MARC-145 cells (Fig 3F–3I). The TG level was higher in sh*RAB18*-1 cells than in control cells during PRRSV-2 infection (Fig 3H). However, there was a defective upregulation of NEFA in cells with RAB18 knockdown (Fig 3I), suggesting that RAB18 deficiency inhibited PRRSV-2-induced lipolysis. We also analyzed the expression of lipases such as hormone-sensitive lipase (HSL) and adipose triglyceride lipase (ATGL). During PRRSV-2 infection, HSL was increased in both sgcontrol and sg*RAB18* MARC-145 cells, while ATGL and PRRSV-2-N expression was decreased in *RAB18* null cells (Fig 3J), suggesting ATGL was critical for RAB18-mediated lipolysis and viral replication.

Given that LDs are involved in virus assembly [28, 29], we then observed PRRSV-2 assembly. A significant decrease in PRRSV-2 particle production was observed in sh*RAB18*-1 cells (Fig 3K), indicating that silencing *RAB18* expression affected PRRSV-2 assembly. Meanwhile, we also analyzed the viral life cycle in scramble and sh*RAB18*-1 MARC-145 cells. Our data indicated that knockdown of *RAB18* did not affect viral attachment and entry (S2 Fig). Taken together, our data demonstrate that RAB18 is responsible for PRRSV-2-induced lipolysis, and viral replication and assembly.

### RAB18 and lysosome are essential for PLIN2 degradation

**Fig 4:**
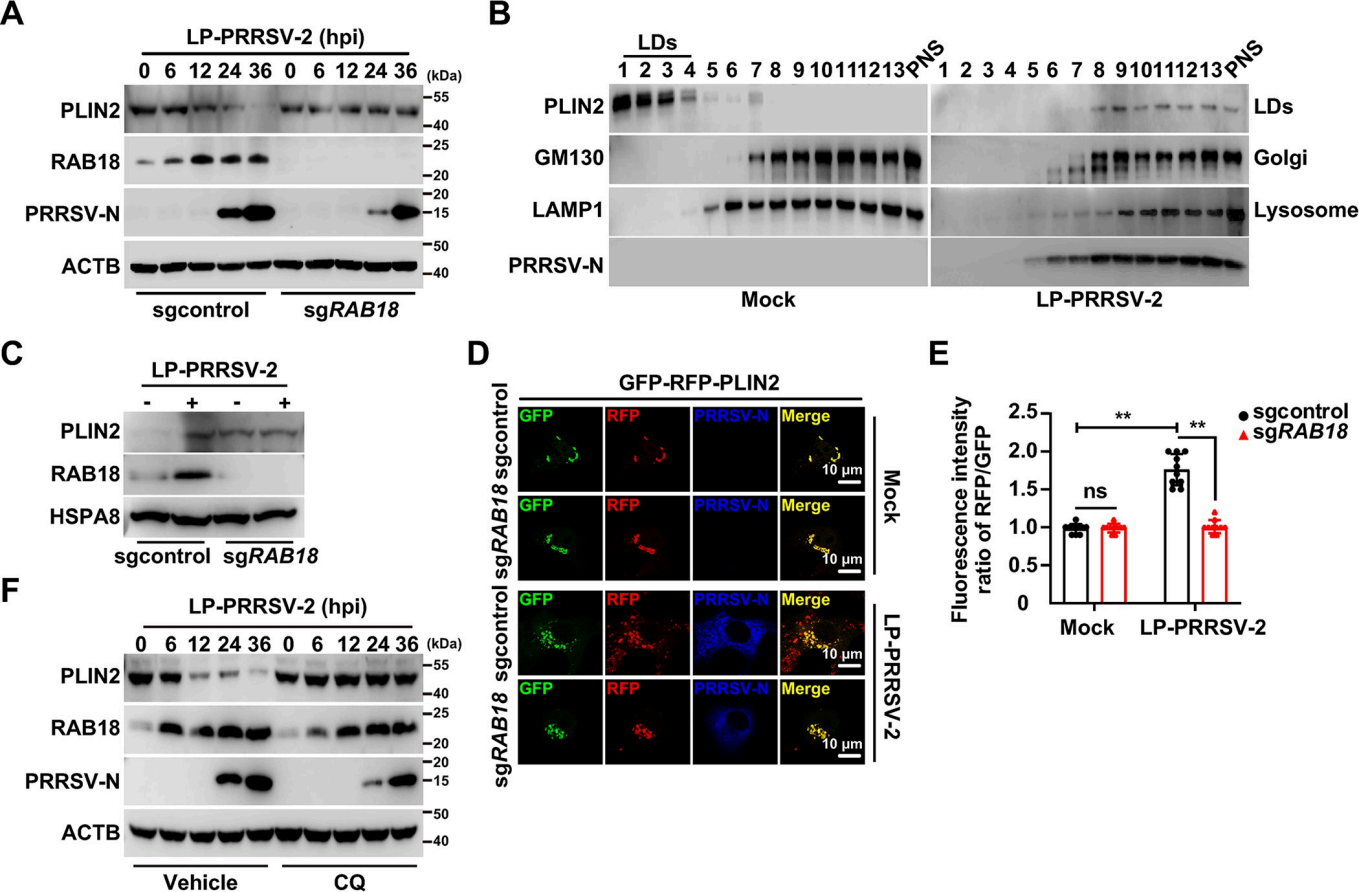
PRRSV-2 infection-induced PLIN2 degradation requires RAB18 and lysosome. (A) Sgcontrol and sg*RAB18* MARC-145 cells were infected with LP-PRRSV-2 (MOI = 10) for 0–36 h. PLIN2, RAB18, PRRSV-2-N and ATCB were analyzed by immunoblot analysis. (B) MARC-145 cells were mock-infected or infected with LP-PRRSV-2 (MOI = 10) for 36 h. Immunoblot analysis of PLIN2 (LDs), GM130 (Golgi), LAMP1 (Lysosome) and PRRSV-2-N was performed with cell lysates subjected to iodixanol density gradient centrifugation. (C) LDs were purified from MARC-145 cells mock-infected or infected with LP-PRRSV-2 (MOI = 10) for 36 h. PLIN2, RAB18 and PRRSV-2-N were analyzed by immunoblot analysis. (D) Sgcontrol and sg*RAB18* MARC-145 cells were transfected with a plasmid encoding GFP-RFP-PLIN2 for 12 h and then mock-infected or infected with LP-PRRSV-2 (MOI = 10) for 36 h. PRRSV-2-N was detected by immunofluorescence analysis and the fluorescence of GFP and RFP was monitored by fluorescence microscopy. Scale bar: 10 μm. (E) Quantification of the fluorescence intensity ratio of RFP/GFP from D by ImageJ (n = 10). **P < 0.01. ns, no significance. (F) MARC-145 cells were mock-infected or infected with LP-PRRSV-2 (MOI = 10), and treated with vehicle or CQ (10 mM) for 0–36 h. PLIN2, RAB18, PRRSV-2-N and ATCB were analyzed by immunoblot analysis.

Perilipin 2 (PLIN2) is a constitutive LDs protein and protects LDs from lipolysis [30], therefore, we examined whether PRRSV-2 infection stimulated PLIN2 degradation through RAB18. Immunoblot analysis indicated that PRRSV-2 infection promoted PLIN2 degradation in sgcontrol cells, but not in sg*RAB18* cells (Fig 4A). This result suggested that RAB18 was essential for PLIN2 degradation during PRRSV-2 infection. We further purified LDs from MARC-145 cells that were either mock-infected or infected with LP-PRRSV-2 for 36 hours. Immunoblot analysis showed that PRRSV-2 infection resulted in the redistribution of PLIN2 from the LDs fraction to those of the Golgi and lysosome (Fig 4B). Additionally, we observed an accumulation of PLIN2 and RAB18 in LDs from control cells compared to those from sg*RAB18* cells in response to PRRSV-2 infection (Fig 4C). These results suggest that PLIN2 is degraded in dependent of RAB18 during PRRSV-2 infection.

Since our data indicated that PLIN2 was redistributed to the fractions of the Golgi and lysosome (Fig 4B), we hypothesized that RAB18 mediated the lysosomal degradation of PLIN2. To test this hypothesis, we first generated a sensor protein with tandem fluorescent tags on PLIN2 (GFP-RFP-PLIN2). When this sensor protein undergoes degradation in the lysosome, the acidic and proteolytic environment of the lysosome quenches the GFP signal due to its pH sensitivity, while the RFP fluorescence remains visible because it is more stable under acidic conditions. We observed that the fluorescence intensity ratio of RFP to GFP was comparable in both mock-infected sgcontrol and sg*RAB18* cells, and in PRRSV-2-infected sg*RAB18* MARC-145 cells (Fig 4D and 4E). In the control cells, PRRSV-2 infection induced a greater increase in RFP signal compared to GFP, a trend not seen in sg*RAB18* MARC-145 cells (Fig 4D and 4E), suggesting that PLIN2 degradation occurred in the lysosome. In addition, we inhibited lysosomal function with chloroquine (CQ), which showed an inhibitory effect on PLIN2 degradation in PRRSV-2-infected MARC-145 cells (Fig 4F). Taken together, our data suggest that PLIN2 degradation is RAB18- and lysosome-dependent during PRRSV-2 infection.

### Rab18 is a crucial mediator of PRRSV-2-triggered CMA

**Fig 5:**
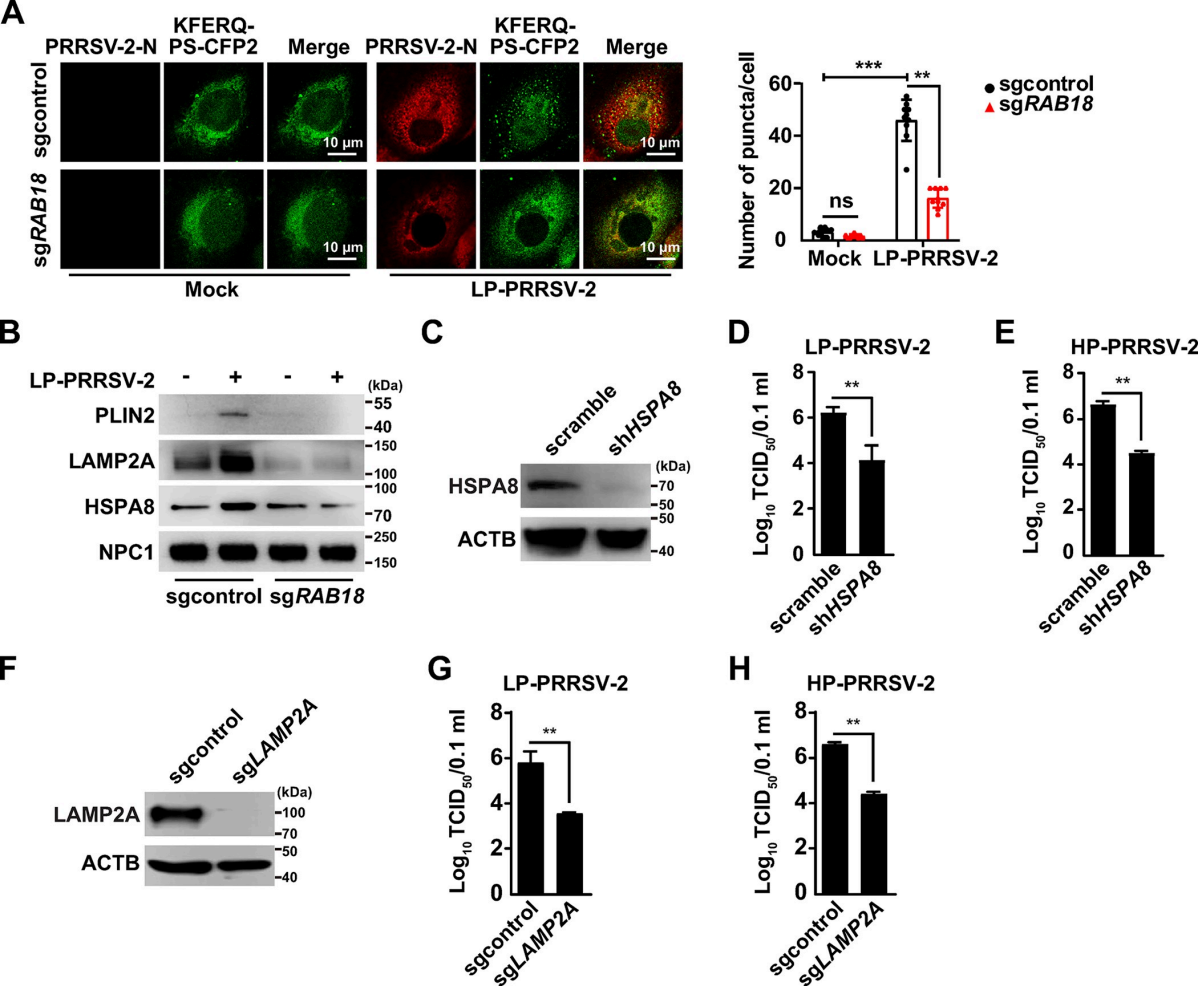
RAB18-mediated CMA facilitates the yield of PRRSV-2 progeny virus. (A) Sgcontrol and sg*RAB18* MARC-145 cells stably expressing KFERQ-PS-CFP2 were photoconverted and maintained for 2 h, and then mock-infected or infected with LP-PRRSV-2 (MOI = 10) for 36 h. The fluorescence of KFERQ-PS-CFP2 was detected by fluorescence microscopy (left). Quantification of the KFERQ puncta per cell by ImageJ is shown on the right (n = 10). Scale bar: 10 μm. **P < 0.01, ***P < 0.001. ns, no significance. (B) Sgcontrol and sg*RAB18* MARC-145 cells were mock-infected or infected with LP-PRRSV-2 (MOI = 10) for 36 h. PLIN2, LAMP2A, HSPA8, and NPC1 in lysosome purified by iodixanol density gradient centrifugation were analyzed by immunoblot analysis. (C) Immunoblot analysis of HSPA8 and ACTB in scramble and sh*HSPA8* MARC-145 cells. (D and E) Scramble and sh*HSPA8* MARC-145 cells were infected with LP-PRRSV-2 (MOI = 10, D) and HP-PRRSV-2 (MOI = 10, E) for 36 h. Viral titers were assessed by the TCID_50_ assay. **P < 0.01. (F) Immunoblot analysis of LAMP2A and ACTB in scramble and sh*LAMP2A* MARC-145 cells. (G and H) Scramble and sh*LAMP2A* MARC-145 cells were infected with LP-PRRSV-2 (MOI = 10, G) and HP-PRRSV-2 (MOI = 10, H) for 36 h. Viral titers were assessed by the TCID_50_ assay. **P < 0.01.

It has been suggested that the degradation of LDs-associated proteins by CMA facilitates lipolysis [21]. To verify that PRRSV-2 infection induces CMA, we employed a photoconversion-based fluorescence technique named KFERQ-PS-CFP2 to observe and quantify CMA activity in living cells [31]. In this assay, the KFERQ motif directs the cyan fluorescent protein CFP2 to the lysosome specifically via CMA. When CMA is active, CFP2 accumulates into puncta within lysosome, and its fluorescence can be detected and quantified using fluorescence microscopy. The green fluorescent pattern of KFERQ-PS-CFP2, which was predominantly diffuse, shifted to a punctate form following PRRSV-2 infection (Fig 5A). Quantification revealed a significant increase in the number of fluorescent KFERQ-PS-CFP2 puncta per cell in PRRSV-2-infected control cells compared to sg*RAB18* cells, which did not show an increase (Fig 5A).

As an alternative approach to determine CMA activation, we measured CMA activity *in vitro* upon isolation of lysosome. HSPA8 is the key chaperone protein in CMA [32]. Immunoblot analysis showed that both PLIN2 and HSPA8 were increasingly distributed to lysosome during PRRSV-2 infection, a phenomenon not observed in sg*RAB18* cells (Fig 5B). These results suggested that PRRSV-2 infection indeed induces CMA. Furthermore, we analyzed whether CMA was involved in the yield of PRRSV-2 progeny virus by using shRNA-mediated knockdown of *HSPA8* and *LAMP2A*. As shown in Fig 5C–5H, the knockdown of either *HSPA8* or *LAMP2A* significantly inhibited the replication of both low and high pathogenic strains of PRRSV-2. Collectively, our data demonstrate that PRRSV-2 hijacks CMA-mediated lipolysis through RAB18.

### HSPA8 facilitates RAB18-PLIN2 interaction

**Fig 6:**
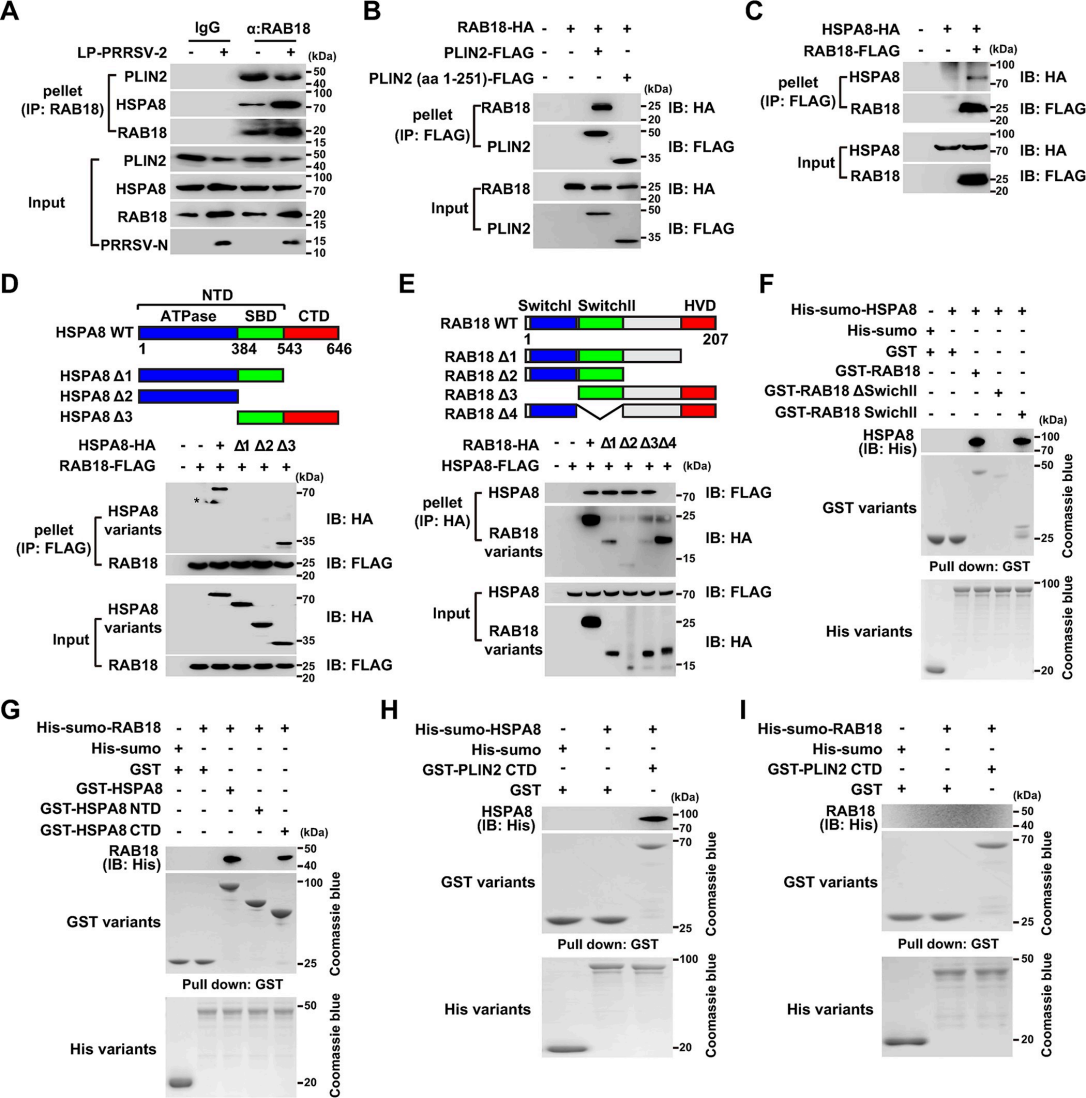
HSPA8 facilitates the association of RAB18 with PLIN2. (A) MARC-145 cells were mock-infected or infected with LP-PRRSV-2 (MOI = 10) for 36 h. The interaction of RAB18, PLIN2, and HSPA8 was analyzed by Co-IP analysis with IgG (control) or anti-RAB18 antibody. (B) HEK293T cells were co-transfected with Rab18-HA and PLIN2-FLAG or PLIN2 (aa 1–251)-FLAG for 24 h. The interaction of RAB18 with PLIN2 variants was analyzed by Co-IP analysis. (C) HEK293T cells were co-transfected with HSPA8-HA and RAB18-FLAG for 24 h. The interaction of HSPA8 with RAB18 was analyzed by Co-IP analysis. (D) HEK293T cells were co-transfected with RAB18-FLAG and indicated HSPA8-HA variants for 24 h. The interaction of RAB18 with HSPA8 variants was analyzed by Co-IP analysis. (E) HEK293T cells were co-transfected with HSPA8-HA and indicated RAB18-FLAG variants for 24 h. The interaction of HSPA8 with RAB18 variants was analyzed by Co-IP analysis. (F) The interaction of His-SUMO-HSPA8 with indicated GST-RAB18 variants was assessed by *in vitro* affinity isolation assays. (G) The interaction of His-SUMO-RAB18 with indicated GST-HSPA8 variants was assessed by *in vitro* affinity isolation assays. (H) The interaction of His-SUMO-HSPA8 with GST-PLIN2 CTD was assessed by *in vitro* affinity isolation assays. (I) The interaction of His-SUMO-RAB18 with GST-PLIN2 CTD was assessed by *in vitro* affinity isolation assays.

To reveal the role of RAB18 in PRRSV-2-induced CMA, we performed co-immunoprecipitation (Co-IP) analysis to analyze the interactions among RAB18, HSPA8, and PLIN2. As shown in Fig 6A, these three proteins interacted with each other in mock-infected cells, and PRRSV-2 infection enhanced their interactions. Co-IP analysis of RAB18 and PLIN2 was also performed in HEK293T cells. A significant amount of PLIN2-Flag was co-precipitated with RAB18-HA, whereas the truncation mutant PLIN2 (1–251)-Flag was not (Fig 6B). On the other hand, RAB18 was also found to associate with HSPA8 (Fig 6C). These results suggest that RAB18, HSPA8, and PLIN2 form a complex in CMA.

To better understand how RAB18 interacted with HSPA8, we generated truncation mutants of both RAB18 and HSPA8. HEK293T cells were transfected with truncation mutants of RAB18-Flag and HSPA8-HA (Fig 6D). Co-IP analysis indicated that the deletion of the ATPase domain of HSPA8 had no impact on its interaction with RAB18. However, HSPA8 lacking the CTD (C-terminal domain), or both the CTD and the SBD (substrate-binding domain), was unable to interact with RAB18 (Fig 6D). This result suggests that the CTD of HSPA8 is essential for its interaction with RAB18. Subsequently, HEK293T cells were transfected with HSPA8-Flag and RAB18-HA truncation mutants (Fig 6E). It was found that HSPA8 failed to co-precipitate with RAB18 lacking the Switch II domain (Fig 6E), suggesting that the Switch II domain of RAB18 mediates its interaction with HSPA8.

Furthermore, we purified the recombinant proteins RAB18, HSPA8, and PLIN2 and performed *in vitro* affinity-isolation assays. HSPA8 directly associated with RAB18 and its Switch II domain; however, deletion of this domain prevented RAB18 from interacting with HSPA8 (Fig 6F). HSPA8 and its CTD bound to RAB18, but not the N-terminal domain of HSPA8 (Fig 6G). Conversely, HSPA8 interacted with the CTD of PLIN2 (Fig 6H), but RAB18 did not interact (Fig 6I). Taken together, our data indicate that HSPA8 serves as a bridge between RAB18 and PLIN2 in lipolysis mediated by CMA.

## Discussion

**Fig 7:**
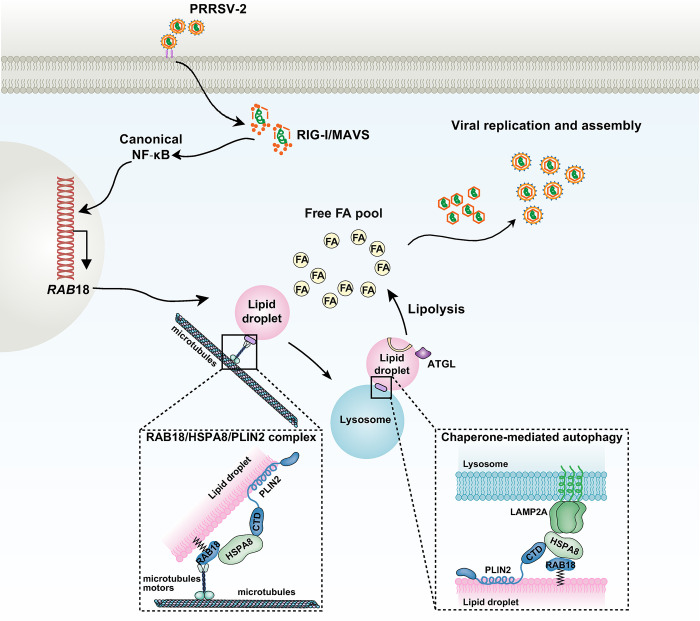
Schematic model of PRRSV-2-induced RAB18 upregulation and CMA-Mediated lipolysis. PRRSV-2 infection activates the RIG-I/MAVS and canonical NF-κB pathways, which in turn upregulate RAB18 expression. RAB18 recruits PLIN2 through direct interaction with HSPA8 and thereby translocates PLIN2 to the lysosome for CMA-mediated degradation. Neutral lipases, such as ATGL, stimulate lipolysis, facilitating PRRSV-2 replication and assembly.

Many pathogens have evolved specific mechanisms to modulate or hijack the dynamics and trafficking functions of RAB GTPases for viral replication [13]. In this study, we found that RAB18 was critical for PRRSV-2 replication through shRNA screening of a targeted library of 62 RABs. PRRSV-2 infection activated the innate immune RIG-I/MAVS and canonical NF-κB signaling pathways, which upregulated RAB18 expression. This, in turn, led to the recruitment of PLIN2 through the CMA chaperone HSPA8. The RAB18/HSPA8/PLIN2 complex was transported to the lysosome for CMA-mediated degradation of PLIN2, followed by subsequent lipolysis that promoted PRRSV-2 replication and assembly (Fig 7).

Proteomic analysis in mammalian systems has revealed the association of over 30 RAB proteins with LDs. Among them, RAB18 has been extensively studied, and there is substantial evidence supporting its direct association with the monolayer surface of LDs [33]. Our library of 62 shRNA-targeted RABs found that RAB18 was indispensable for PRRSV-2 replication. RAB guanine nucleotide exchange factors (GEFs) and GTPase-activating proteins (GAPs) are two distinct classes of proteins that regulate the activity of RAB GTPases [34]. GEFs catalyze the exchange of GDP for GTP on RAB proteins, which activates them, allowing them to interact with downstream effectors [35]. GAPs, on the other hand, stimulate the intrinsic GTPase activity of RABs, leading to their inactivation [36]. Cytokinesis 11, known as a GEF for cell division cycle 42, has been reported to be essential for the maintenance of hepatitis B virus [37]. *In vitro* studies indicate that RAB3GAP1 and RAB3GAP2 combine to form a binary complex known as ’RAB3GAP’, which acts as a GEF for RAB18 [38]. Additionally, TBC1D20 displays modest RAB18 GAP activity [39]. Mutations in RAB18, RAB3GAP1, RAB3GAP2, and TBC1D20 have caused Warburg micro syndrome, a rare autosomal recessive multisystem disorder [40], suggesting that RAB3GAP1, RAB3GAP2, and TBC1D20 might play roles in PRRSV-2 replication. Furthermore, we found that the RAB18/HSPA8/PLIN2 complex was transported to the lysosome for CMA-mediated degradation of PLIN2. Microtubule-dependent motors, such as kinesins or dyneins, participate in the vesicular transport through the cytoplasm toward their target membranes [41]. Although we did not identify the microtubules motors of RAB18, an interesting area for further research would be to better understand the RAB18 effectors in CMA.

RIG-I is the first line of defense against RNA viruses, serving as a pattern recognition receptor that identifies molecular features common to both dsRNA and ssRNA viral pathogens [25]. Our data indicated that disruption of RIG-I, MAVS, and RELA expression prevented the PRRSV-2-induced upregulation of RAB18, suggesting that PRRSV-2 infection upregulated RAB18 expression through the innate immune RIG-I/MAVS and the canonical NF-κB signaling pathways. Subsequently, RAB18 facilitated CMA-mediated lipolysis to assist PRRSV-2 assembly. Our data revealed how PRRSV-2 hijacked CMA-mediated LDs metabolism through innate immune activation to facilitate the yield of progeny virus. It cannot be categorically concluded that PRRSV-2 activates RAB18 expression only via the RIG-I pathway, because other RNA viruses may also stimulate RAB18 expression through the RIG-I/MAVS/NF-κB pathway. However, whether RAB18-mediated CMA is critical for their replication requires further investigation.

It has been reported that the NF-κB signaling pathway contributes to inflammation and metabolic disease [42]. The NF-κB axis promotes liver steatosis by stimulating de novo lipogenesis and cholesterol synthesis [43]. Atherosclerosis is a persistent inflammatory condition of the arterial wall characterized by lipid-filled lesions, which result from the intricate interplay of various cell types and cytokine networks [44]. NF-κB activation is a pathological mechanism of lipid metabolism and atherosclerosis [45]. NF-κB is also important for tumor necrosis factor-alpha-induced lipolysis in human adipocytes [46]. Our data suggest that RAB18-mediated CMA could be involved in tumor necrosis factor-alpha-induced lipolysis.

Our previous and present studies indicate that PRRSV-2 infection promotes LDs biogenesis [47]. It has been reported that RAB18 participates in LDs metabolism [48, 49]. Our data also suggest that more LDs accumulate in cells with RAB18 knockdown than in control cells, due to aberrant LDs catabolism during PRRSV-2 infection. Whether RAB18 is involved in PRRSV-2-induced LDs biogenesis has not been determined. LDs arise from the endoplasmic reticulum (ER) [50]. RAB18 promotes LDs growth by tethering the ER to LDs through soluble N-ethylmaleimide-sensitive factor attachment protein receptor and NAG-RINT1-ZW10 interactions [26]. However, RAB18 appears to be dispensable for LDs maturation and function in several examined cell lines, including HeLa, Cos7, HEK293, and a human mammary carcinoma cell line [26, 51]. Since different viruses exhibit diverse adaptations to various cell types, further investigation is needed to determine whether RAB18 hijacking of CMA-mediated lipolysis is specific to PRRSV-2 or a mechanism common to other viruses. Additionally, we have shown that knockdown of RAB7A also inhibits PRRSV-2 replication. Because RAB7 regulates autophagosome-lysosome fusion [52], we hypothesize that RAB7 coordinates with RAB18 in CMA-mediated lipolysis during PRRSV-2 infection. Whether the reported finding applies also to PRRSV-1 infections remains to be studied.

## Materials and methods

### Ethics statement

Experiments involving animals were approved by the Committee on the Ethics of Animal Care and Use of Henan Agricultural University (HNND2019031004). The study was conducted in accordance with the Guide for the Care and Use of Animals in Research of the People’s Republic of China.

### Viruses

Two Betaarterivirus *suid* 2 isolates low-pathogenic PRRSV-2 strain (LP-PRRSV-2 BJ-4, accession number AF331831), high-pathogenic PRRSV-2 strain (HP-PRRSV-2 HN07-1, accession number KX766378.1, provide by Professor Gai-Ping Zhang from Henan Agricultural University), and the recombinant PRRSV-2-GFP were used as described previously [47].

### Pigs

Five-week-old specific-pathogen-free weaned piglets were intranasally challenged with LP-PRRSV-2 (2 × 10^5^ TCID_50_/piglet) and were euthanized at 30 days post-infection. Lung tissues were collected for immunofluorescence analysis and Oil Red O staining. Animal protocols were performed in accordance with the Guide for the Care and Use of Laboratory Animals and the related ethical regulations at Henan Agricultural University.

### Antibodies and reagents

The antibodies anti-RAB18 (11308-1-AP), anti-PLIN2 (15294-1-AP), anti-HSPA8 (10654-1-AP), and anti-NPC1 (13926-1-AP) were purchased from Proteintech; anti-LAMP1 (#9091), anti-GM130 (#12480), and anti-RELA (#3033) were purchased from Cell Signaling Technology; anti-LAMP2A (ab18528) was purchased from Abcam; anti-HA (A01244) and anti-His_6_ (C157) were purchased from Genscript; anti-FLAG (F1804) and anti-ATCB (A1978) were purchased from Sigma-Aldrich; anti-PRRSV-2 nucleocapsid (N) (SDOW17) was purchased from Rural Technologies; horseradish peroxidase (HRP)-conjugated donkey anti-mouse IgG (715-035-150) and anti-rabbit IgG (711-035-152) antibodies were purchased from Jackson ImmunoResearch Laboratories; anti-rabbit IgG antibodies labeled with Alexa Fluor 488 (A11034) and Alexa Fluor 568 (A11011) and anti-mouse IgG antibodies labeled with Alexa Fluor 488 (A11001), Alexa Fluor 568 (A11004), Alexa Fluor 405 (A31553), and Alexa Fluor Plus 647 (A32728) were purchased from Thermo Fisher Scientific. The antibodies described above were used at dilutions of 1:500 for immunofluorescence and 1:1,000 for immunoblot analysis.

Oil Red O (O0625) was purchased from Sigma-Aldrich. Chloroquine (HY-17589A) was purchased from MedChemExpress. BODIPY 493/503 (D3922) and DAPI (D1306) were purchased from Thermo Fisher Scientific.

### Cells

PAMs were collected from lung lavage samples obtained from 4-week-old specific-pathogen-free piglets (free of PRRSV-1, PRRSV-2, pseudorabies virus, porcine circovirus, and classical swine fever virus). PAMs were cultured in RPMI 1640 (GIBCO, 61870036) supplemented with 10% fetal bovine serum (FBS) (GIBCO, 10099141C), 100 units/mL penicillin, and 100 μg/mL streptomycin sulfate (Sangon, B540732). MARC-145 cells (American Type Culture Collection, ATCC, CRL-12231) and HEK293T cells (ATCC, CRL-11268) were cultivated as previously described [47]. All cells were grown in monolayer cultures at 37°C under 5% CO_2_.

### Plasmids and transfection

The plasmids encoding FLAG-RAB18 and HA-RAB18 were generated by inserting the RAB18 coding sequence into 3×FLAG-CMV-10 or pCAGGS-HA using EcoRI (NEB, #R3101L) and BamHI (NEB, #R3136L). FLAG-RAB18 S22N and FLAG-RAB18 Q67L mutants were created via site-directed mutagenesis (Stratagene, #200518). HSPA8-FLAG and HA-HSPA8 were produced by inserting the HSPA8 coding sequence into 3×FLAG-CMV-10 or pCAGGS-HA using *Eco*RI and *Kpn*I (NEB, #R3142L). Plasmids for RAB18 and HSPA8 truncations were assembled employing standard molecular biology techniques. PLIN2-HA was obtained by inserting the PLIN2 coding sequence into pCAGGS-HA with *Eco*RI and *Kpn*I. GFP-RPF-PLIN2 was created by replacing the LC3 sequence in GFP-RPF-LC3 [47] with that of PLIN2. KFERQ-PS-CFP2 was synthesized by GenScript and cloned into the pLentiCMV-Blast vector (Addgene, #125133). All plasmids were introduced into cells using Lipofectamine 3000 (Invitrogen, #L3000015) according to the manufacturer’s instructions. RAB18, HSPA8, and PLIN2 variants were subcloned into pGEX-4T-3 or pET21b-sumo vectors for prokaryotic expression.

For the luciferase reporter assays, the *RAB18* promoter (1831 bp upstream of the transcription initiation site, +1) and NF-κB binding site mutants were cloned from MARC-145 genomic DNA into pGL3-Basic (Promega, E1751) using the *Kpn*I and *Hind*III restriction sites. This process generated the RAB18-LUC plasmids employing standard molecular biology techniques. pCMV-Renilla was used as previously described [53].

### Cell viability assay

Cell viability was evaluated using a Cell Counting Kit-8 (CCK-8) according to the manufacturer’s instructions (Dingguo, GK3607). Briefly, cells were seeded in 96-well plates at a concentration of 8 × 10^3^ cells per well. CCK-8 solution (10 μL) was added to each well at specific time points, and the cells were incubated at 37°C for an additional 3 hours. The absorbance at 450 nm was measured using a microplate reader (VARIOSKAN FLASH, Thermo Fisher Scientific).

### Flow cytometry assay

MARC-145 cells were infected with PRRSV-2-GFP (MOI = 10) for 36 h. The cells were digested with trypsin-EDTA (GIBCO, 25200072), collected by centrifugation, and resuspended in phosphate-buffered saline (PBS). The percentage of GFP-positive cells was measured by flow cytometry using a CytoFLEX instrument.

### Immunoblot analysis

Cells were collected in lysis buffer (50 mM Tris-HCl, pH 8.0; 150 mM NaCl; 1% Triton X-100; 1% sodium deoxycholate; 0.1% SDS; and 2 mM MgCl_2_) supplemented with a protease and phosphatase inhibitor cocktail (MedChemExpress, HY-K0010 and HY-K0022). The protein concentrations of the lysates were quantified with a BCA Protein Assay Kit (Dingguo Biotechnology, BCA01). Protein samples were separated by SDS-PAGE and transferred to membranes (Millipore, ISEQ00010), which were then incubated in 5% nonfat milk (Sangon, A600669) at room temperature for 1 hour. The membranes were incubated with primary antibodies overnight at 4°C and subsequently with appropriate secondary antibodies for 1 hour. Immunoblot results were visualized using Luminata Crescendo Western HRP Substrate (Millipore, Catalog No. WBLUR0500) on a GE AI600 imaging system.

### Immunofluorescence analysis

Cells grown on coverslips (Thermo Fisher Scientific, 12-545-80) were fixed with 4% paraformaldehyde for 30 minutes, permeabilized with 0.1% Triton X-100, and then incubated with PBS containing 10% FBS (10% FBS in PBS) and the primary antibody for 1 hour at room temperature. The cells were washed three times with PBS and subsequently labeled with the fluorescent secondary antibody in 10% FBS/PBS for 1 hour. Finally, the cells were washed in PBS and mounted in ProLong Diamond with DAPI (Invitrogen, P36971). Images were captured using a Zeiss LSM 800 confocal microscope and processed with ImageJ software for quantitative image analysis.

### Immunohistochemistry

Lung tissues were washed in PBS, fixed in 4% paraformaldehyde for 4 hours at 4°C, and then cut into 4-μm-thick paraffin sections. Before antibody staining, the sections were deparaffinized in xylene, rehydrated through a graded ethanol series, and subsequently subjected to high-temperature antigen retrieval in 50 mM Tris-HCl (pH 9.0) and 1 mM EDTA. Sections were blocked and permeabilized in PBS containing 0.5% Triton X-100 and 5% FBS. Primary antibodies from different species were co-incubated during the staining procedure. Sections were then washed and further blocked with mouse and rabbit IgG (Proteintech, B900620 and 30000-0-AP) for 30 minutes before incubation with an Alexa Fluor 488-labeled mouse anti-PRRSV-2-N antibody and an Alexa Fluor 568-labeled rabbit anti-RAB18 antibody. After the immunoreaction, sections were incubated with Hoechst for 5 minutes before mounting. Slides were observed using an Olympus BX53 microscope.

### qRT-PCR analysis

Total RNA was isolated with TRIzol Reagent (TaKaRa, 9108) and subjected to cDNA synthesis with a PrimeScript RT reagent Kit (TaKaRa, RR047A). The qRT-PCR was performed in triplicate by using SYBR Premix Ex Taq (TaKaRa, RR820A), according to the manufacturer’s instructions, and data were normalized to the level of *ATCB* expression in each sample. Melting curve analysis indicated the formation of a single product in all cases. The 2^−ΔΔCt^ method was used to calculate relative expression changes. Primers used for qRT-PCR are shown in **S1 Table**.

### RNAi

Lentivirus-mediated RNAi was conducted as previously described [47]. Briefly, shRNAs were synthesized as double-strand oligonucleotides, cloned into the pLKO.1 vector (Sigma-Aldrich, SHC001), and co-transfected with packaging plasmids pMD2.G and psPAX2 (Addgene, #12259 and #12260) into HE293T cells. Lentiviruses were harvested at 48 h post-transfection and used to infect cells that were then selected with puromycin (4 μg/mL; Solarbio, P8230) for 7 days. Knockdown efficiency was determined by qRT-PCR or immunoblot analysis. The shRNA sequences used for RNAi are shown in **S2 Table**.

The siRNA-mediated RNAi was performed as described previously [54]. Cells were seeded in 60-mm dishes at a density of 4 × 10^5^ cells per dish and were transfected with siRNA (GenePharma, Shanghai) at a final concentration of 0.12 nM. Transfections were performed with Lipofectamine RNAiMAX Reagent (Invitrogen, 13778500), according to the manufacturer’s instructions in Opti-MEM reduced serum medium (GIBCO, 31985062). The medium was replaced with DMEM containing 10% FBS at 8 h post-transfection. The knockdown efficacy was assessed by qRT-PCR or immunoblot at 48 h post-transfection. The siRNA sequences used for RNAi are shown in **S2 Table**.

### Dual-luciferase reporter assays

Cells cultured in 24-well plates were co-transfected with pCMV-Renilla (normalization plasmid) and RAB18–Luc (luciferase reporter plasmid) variants with Lipofectamine 3000. At 24 h post-transfection, luciferase reporter assays were performed with the Dual-Luciferase Reporter Assay System (Promega, E1910), according to the manufacturer’s instructions. The luminescence signal was detected with a Fluoroskan Ascent FL Microplate Fluorometer (Thermo Fisher Scientific).

### Generation of gene knockout cell lines via CRISPR/Cas9

Small guide RNAs (sgRNAs) targeting *RAB18* and *LAMP2A* (sg*RAB*18: 5′-TGAAGGGCTGAGAGGCGCAC-3′; sg*LAMP2A*: 5′-GCTTCCCGGTTCCGGGCTCA-3′) were synthesized and cloned into the lentiCRISPR v2 vector (Addgene, #52961), according to the manufacturer’s instructions. Lentiviral production was the same as in the method of lentivirus-mediated RNAi. MARC-145 cells were infected with lentiviruses and then selected with puromycin (4 μg/mL) for 7 days. Single clonal knockout cells were obtained by serial dilution and verified by Sanger DNA sequencing and immunoblot analysis.

### ChIP

Cells grown in 10 cm dishes were cross-linked with DMEM containing 1% formaldehyde (Sigma-Aldrich, 158127) for 15 min, and then the crosslinking was stopped by the addition of 125 mM glycine for 5 min. After being washed twice with PBS, the cells were incubated in lysis buffer (10 mM Tris-HCl, pH 7.5, 10 mM KCl, 5 mM MgCl_2_, and 0.5% NP40) supplemented with protease inhibitor cocktail on ice for 10 min and centrifuged at 380 × *g* for 5 min. The cell pellets containing chromatin were suspended in SDS lysis buffer (50 mM Tris-HCl, pH 7.9, 10 mM EDTA, and 0.5% SDS) supplemented with a protease inhibitor cocktail and sonicated into fragments with an average length of 1 kb. ChIP assays were performed with IgG or antibody against RELA. Primers specific for *RAB18* promoter were as follows:

primer-1-Fw: 5′-AGACCTGAAGGAGCTGAAGC-3′ and primer-1-Rv: 5′-TGTACCTGCAGCTAATGTCAT-3′;

primer-2-Fw: 5′-GAATGGACAGGGAAGGCCTC-3′ and primer-2-Rv: 5′-CTGGAAAAGCCCTTTCAAAAGA-3′.

### Co-IP

Cells were transfected with the indicated plasmids for 24 h. Cells were then harvested and lysed in 1 mL of lysis buffer (PBS, 1% NP-40, 5 mM EDTA, and 5 mM EGTA) and clarified by centrifugation at 16,500 × *g* for 10 min at 4°C. Next, 900 μL aliquots were incubated with 40 μL of a 1:1 slurry of Sepharose conjugated with either IgG (GE Healthcare, 17-0969-01), anti-FLAG mouse mAb (Sigma-Aldrich), or anti-HA mouse mAb (Genscript) for 4 h. The beads were washed four times with lysis buffer and were eluted with SDS sample buffer by boiling for 10 min before immunoblot analysis.

### In vitro affinity isolation assays

GST-tagged and His-tagged recombinant proteins were expressed in *E*.*coli* BL21 and purified under nondenaturing conditions, using BeyoGold GST-tag purification resin (Genscript, P2253) and Ni-NTA agarose (Qiagen, 30210), according to the manufacturer’s instructions. Aliquots of GST-tag purification resin containing 1 nM of the recombinant His-tagged proteins were mixed with 1 nM of each GST fusion protein in 500 μL of binding buffer (PBS+1% Triton X-100) and incubated for 45 min on a rotating platform at 4°C. Then the beads were collected by centrifugation at 1,000 × *g* for 3 min. Supernatants were removed and beads were washed five times with binding buffer. The proteins bound to beads were eluted with SDS sample buffer by boiling for 10 min before immunoblot analysis.

### Lysosome purification by iodixanol density gradient centrifugation

Lysosome were purified with a Lysosome Isolation Kit (Sigma-Aldrich, LYSISO1), according to the manufacturer’s instructions. The homogenate of cells was centrifuged serially at 1,000 × *g* for 10 min and 20,000 × *g* for 20 min. The pellets were collected and placed on 8%, 12%, 16%, 19%, 22.5%, and 27% (v/v) iodixanol gradients, then centrifuged at 150,000 × *g* for 4 h. Fractions (0.8 mL each) were collected from the top to the bottom. The fractions with the purest lysosome content were pooled together.

### LDs isolation

Cells were washed with chilled PBS and subsequently centrifuged to gather them into a pellet. This pellet was resuspended in 10 mL of Buffer A, which consists of 25 mM Tricine at pH 7.8 and 250 mM sucrose, along with 0.5 mM PMSF for protease inhibition. The mixture was then left to stand on ice for 20 minutes, enabling the cells to swell. Afterward, the cells were disrupted using a nitrogen decompression cell disruption bomb at a pressure of 500 psi, for 15 minutes under cold conditions. To eliminate cell debris, the resultant mixture was subjected to a low-speed centrifugation at 3000 × *g* for 10 minutes at a temperature of 4°C. Following this step, 8 mL of the resulting post-nuclear supernatant (PNS) was retrieved and carefully layered into a SW40 centrifuge tube. This was then gently topped with about 3 mL of Buffer B, which has a composition of 20 mM HEPES at pH 7.4, 100 mM KCl, and 2 mM MgCl_2_. The assembly was ultracentrifuged at 250,000 × *g* for one hour at 4°C. The top fraction of the gradient, a white band harboring the LDs, was then carefully transferred into a 1.5 mL Eppendorf tube. To thoroughly clean the LDs, they were washed thrice with Buffer B, each wash followed by centrifugation at 20,000 × *g*, maintaining the temperature consistently at 4°C.

### LDs staining

For Oil Red O staining, cells were washed with PBS and fixed in 4% PFA for 30 min. The cells were washed again with PBS and then stained with Oil Red O (saturated Oil Red O solution in isopropanol-water at a 3:2 dilution) for 15 min. The cells were then washed with 70% alcohol for 5 s to remove the background stain, rinsed in double-distilled water, counterstained with Harris hematoxylin (10 s), washed, mounted, and observed under a light microscope. The LDs number was determined with the ImageJ “analyze particles” function (areas of particles of < 0.01 mm^2^ were excluded). Pig lungs were fixed in 4% paraformaldehyde, incubated in 30% sucrose/PBS (as a cryoprotectant), and frozen in OCT. Frozen tissue was sectioned (20 mm) for Oil Red O staining.

For BODIPY staining, cells were washed with PBS and fixed in 4% PFA for 30 min. After the cells were washed again with PBS, they were incubated with 2 μg/mL BODIPY 493/503 (493-nm excitation/503-nm emission) for 30 min. Digital images were obtained with an LSM 800 confocal microscope. Fluorescence intensity was determined with ImageJ software.

### Photoconvertible CMA reporter assay

CMA activity was measured by using photoswitchable (PS) artificial substrates as described previously [31]. Cells were infected with lentiviruses to generate cells stably expressing KFERQ-PS-CFP2. The cells were photoactivated by exposure to a 405/20 nm LED array (Norlux) for 15 min using 50 mWcm^−2^ light intensity. When exposed to a 405 nm LED light, more than 90% of the KFERQ-PS-CFP2 changes from blue to green fluorescence. Cells were fixed with 4% paraformaldehyde for 30 min and images were captured on a Zeiss LSM 800 confocal microscope. CMA can be quantified as the number of fluorescent puncta per cell by ImageJ software.

### Determination of intracellular TG and NEFA

The cell lysates were extracted with a syringe needle in 250 μL of RIPA buffer and centrifuged at 12,000 × *g* for 5 min at 4°C. The total lipids in 200 μL of the lysate were extracted by the addition of 100 μL of a chloroform-methanol (2:1, v/v) mixture. The extract was evaporated to dryness and dissolved in 50 μL of TRB (100 mM KH_2_PO_4_, 100 mM K_2_HPO_4_, 5 mM sodium cholate, 50 mM NaCl, 0.1% Triton X-100, pH 7.4). The values were normalized to the total cellular protein content, which was determined with a BCA Protein Assay Kit (DINGGUO Biotechnology, BCA01). TG and NEFA were measured with a LabAssay Triglyceride kit (Wako, 290–63701) and a LabAssay NEFA kit (Wako, 294–63601), according to the manufacturer’s instructions.

### Viral titration

MARC-145 cells were seeded in 96-well plates at a density of 1 × 10^4^ cells per well. On the next day, the cells were inoculated with serially diluted viruses (10^−1^–10^−12^ fold) for 1 h at 37°C. The excess viral inoculum was removed by washing with PBS. Then 200 μL of DMEM/2% FBS was added to each well, and the cells were further cultured for 3–5 days. The cells that demonstrated the expected cytopathic effects were observed daily, and the TCID_50_ value was calculated using the Reed-Muench method.

### Statistical analysis

GraphPad Prism 7 software was used for data analysis. Data are shown as mean ± standard deviations from three independent experiments. Statistical significance between two groups was analyzed with a two-tailed unpaired Student’s *t*-test.

## Supporting information

S1 Data Data set. (XLSX)

S1 Table Primers for qRT-PCR. (XLSX)

S2 Table shRNAs and siRNAs for RNAi. (XLSX)

S1 Fig Cell viability assay. (A) Cell viability was assessed by a CCK-8 assay in MARC-145 cells with the indicated RAB knockdown at 72 h. (B) Cell viability was assessed by a CCK-8 assay in scramble, sh*RAB18*-1 and sh*RAB18*-2 MARC-145 cells for 0–72 h. (C) Cell viability was assessed by a CCK-8 assay in sgcontrol and sg*RAB18* MARC-145 cells for 0–72 h. (D) MARC-145 cells were transfected with plasmids encoding RAB18 WT, S22N and Q67L for 0–48 h. Cell viability was assessed by a CCK-8 assay. (E) MARC-145 cells were transfected with the indicated siRNA for 48 h. Cell viability was assessed by a CCK-8 assay. (F) MARC-145 cells were transfected with the indicated siRNA for 24 h and then infected with LP-PRRSV-2 (MOI = 10) for 36 h. Viral titers were assessed by the TCID_50_ assay. *P < 0.05.
(TIF)

S2 Fig RAB18 is not responsible for PRRSV-2 attachment, entry, and replication. (A) Scramble and sh*RAB18*-1 MARC-145 cells were incubated with LP-PRRSV-2 (MOI = 10) at 4°C for 2 hours. After three extensive washes with ice-cold PBS, viral attachment was detected by qRT-PCR analysis of PRRSV-2 ORF7 mRNA on the PM. ns, no significance. (B) Scramble and sh*RAB18*-1 MARC-145 cells were treated as in A. Viral attachment was detected by immunofluorescence analysis of PRRSV-2-N protein on the plasma membrane. Scale bar, 10 μm. (C) Quantification of PRRSV-2-N puncta per field from B. ns, no significance. (D) Scramble and sh*RAB18*-1 MARC-145 cells were incubated with LP-PRRSV-2 (MOI = 10) at 4°C for 2 hours. The cells were then shifted to 37°C for 10 min to allow entry. After washing with trypsin (1 mg/mL) to remove the residual virions on the PM, viral entry was detected by qRT-PCR analysis of PRRSV-2 ORF7 mRNA in the cells. ns, no significance.
(TIF)
