# Supplementary figures and images for: Porcine reproductive and respiratory syndrome virus 2 hijacks CMA-mediated lipolysis through upregulation of small GTPase RAB18
Source: PLoS Pathog. 2024 Apr 12;20(4):e1012123. doi: 10.1371/journal.ppat.1012123 (PMC11014436; doi:10.1371/journal.ppat.1012123)

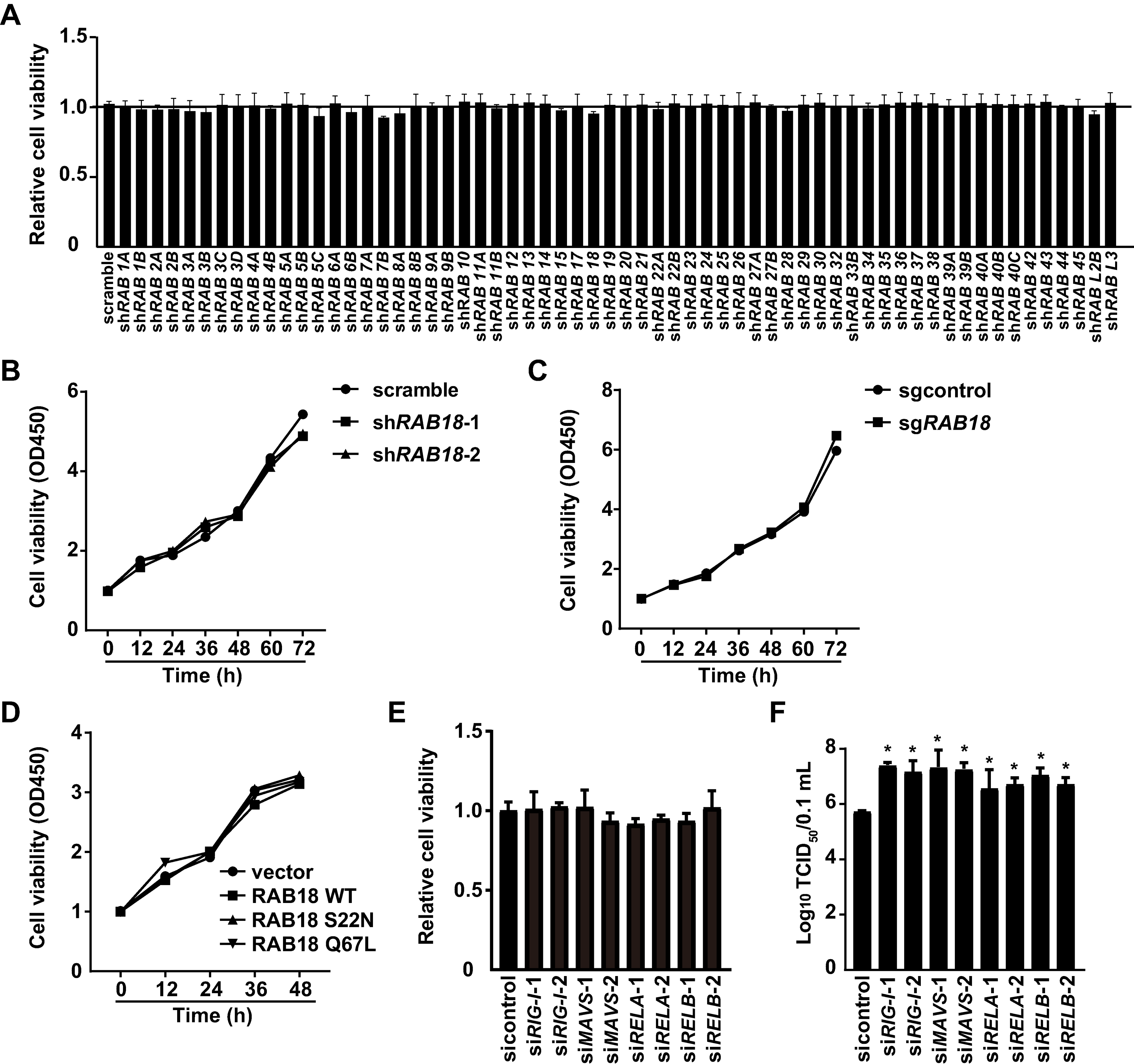

Supplement: S1 Fig — (A) Cell viability was assessed by a CCK-8 assay in MARC-145 cells with the indicated RAB knockdown at 72 h. (B) Cell viability was assessed by a CCK-8 assay in scramble, shRAB18-1 and shRAB18-2 MARC-145 cells for 0–72 h. (C) Cell viability was assessed by a CCK-8 assay in sgcontrol and sgRAB18 MARC-145 cells for 0–72 h. (D) MARC-145 cells were transfected with plasmids encoding RAB18 WT, S22N and Q67L for 0–48 h. Cell viability was assessed by a CCK-8 assay. (E) MARC-145 cells were transfected with the indicated siRNA for 48 h. Cell viability was assessed by a CCK-8 assay. (F) MARC-145 cells were transfected with the indicated siRNA for 24 h and then infected with LP-PRRSV-2 (MOI = 10) for 36 h. Viral titers were assessed by the TCID50 assay. *P < 0.05. (TIF) [file ppat.1012123.s004.tif]

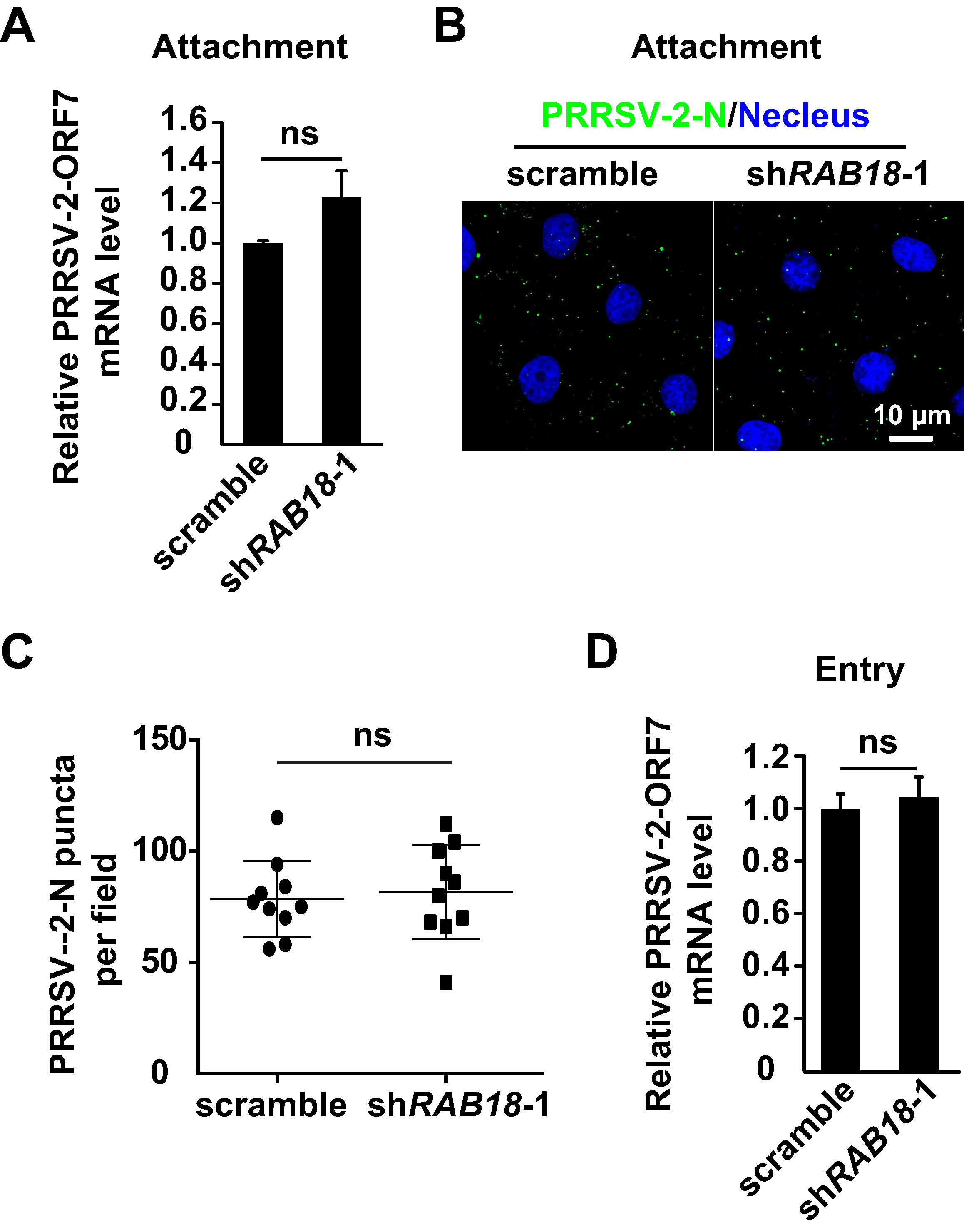

Supplement: S2 Fig — (A) Scramble and shRAB18-1 MARC-145 cells were incubated with LP-PRRSV-2 (MOI = 10) at 4°C for 2 hours. After three extensive washes with ice-cold PBS, viral attachment was detected by qRT-PCR analysis of PRRSV-2 ORF7 mRNA on the PM. ns, no significance. (B) Scramble and shRAB18-1 MARC-145 cells were treated as in A. Viral attachment was detected by immunofluorescence analysis of PRRSV-2-N protein on the plasma membrane. Scale bar, 10 μm. (C) Quantification of PRRSV-2-N puncta per field from B. ns, no significance. (D) Scramble and shRAB18-1 MARC-145 cells were incubated with LP-PRRSV-2 (MOI = 10) at 4°C for 2 hours. The cells were then shifted to 37°C for 10 min to allow entry. After washing with trypsin (1 mg/mL) to remove the residual virions on the PM, viral entry was detected by qRT-PCR analysis of PRRSV-2 ORF7 mRNA in the cells. ns, no significance. (TIF) [file ppat.1012123.s005.tif]
